# Dynamic Pose Estimation Using Multiple RGB-D Cameras

**DOI:** 10.3390/s18113865

**Published:** 2018-11-10

**Authors:** Sungjin Hong, Yejin Kim

**Affiliations:** 1 Creative Content Research Division, Electronics and Telecommunications Research Institute, 218 Gajeong-ro, Yuseong-gu, Daejeon 34129, Korea; sjhong0117@etri.re.kr; 2 School of Games, Hongik University, 2639 Sejong-ro, Jochiwon-eup, Sejong 30016, Korea

**Keywords:** human motion, dynamic pose, body parts detection, motion tracking, depth images, action recognition, motion synthesis

## Abstract

Human poses are difficult to estimate due to the complicated body structure and the self-occlusion problem. In this paper, we introduce a marker-less system for human pose estimation by detecting and tracking key body parts, namely the head, hands, and feet. Given color and depth images captured by multiple red, green, blue, and depth (RGB-D) cameras, our system constructs a graph model with segmented regions from each camera and detects the key body parts as a set of extreme points based on accumulative geodesic distances in the graph. During the search process, local detection using a supervised learning model is utilized to match local body features. A final set of extreme points is selected with a voting scheme and tracked with physical constraints from the unified data received from the multiple cameras. During the tracking process, a Kalman filter-based method is introduced to reduce positional noises and to recover from a failure of tracking extremes. Our system shows an average of 87% accuracy against the commercial system, which outperforms the previous multi-Kinects system, and can be applied to recognize a human action or to synthesize a motion sequence from a few key poses using a small set of extremes as input data.

## 1. Introduction

The detection of human body parts has been popularly researched in the computer vision and pattern recognition fields. Accurate detection of body parts is important in human pose estimation for activity recognition, which is utilized by various smart systems including: Human computer interaction (HCI), surveillance, healthcare, and entertainment. Recently, it has converged with the virtual reality (VR) and augmented reality (AR) techniques in the training field [1].

Early approaches using a single camera tried to detect the region of interest by extracting the features from illumination, color, and edge information on 2D images. In these approaches, machine learning algorithms such as adaptive boosting (AdaBoost), support vector machine (SVM), and gaussian mixture model (GMM) are used to extract key body features such as face, torso, hands, and feet from a large data set. However, a reliable detection of such features is difficult to achieve due to the background noises and illumination changes on the images. The recent availability of red, green, blue, and depth (RGB-D) cameras, such as Microsoft Kinect [2] and Intel RealSense [3] provides depth data and suggests a more reliable way to detect the features. Using depth information retrieved from an infrared sensor, the region of interest on the human body can be segmented more precisely without background ambiguities.

The joints of the human body can provide useful information for motion analysis. Using a single RGB-D camera, the approach introduced by Shotton et al. [4] has been widely used to detect human body parts as a hierarchical skeleton structure. In their approach, a list of joint positions is estimated from a user who faces the RGB-D camera by using the random statistical model. However, its accuracy, especially for internal joints, is sensitive to the input pose. For example, the joint positions of occluded body parts might be either skipped or incorrectly estimated from unknown poses. Using multiple cameras around the user, a set of depth images captured from different viewpoints can be combined to complement the occluded body parts [5,6,7,8,9,10,11]. In these approaches, an optimization problem should be solved to track a list of joints in an articulated model from the depth volume.

**Figure 1:**
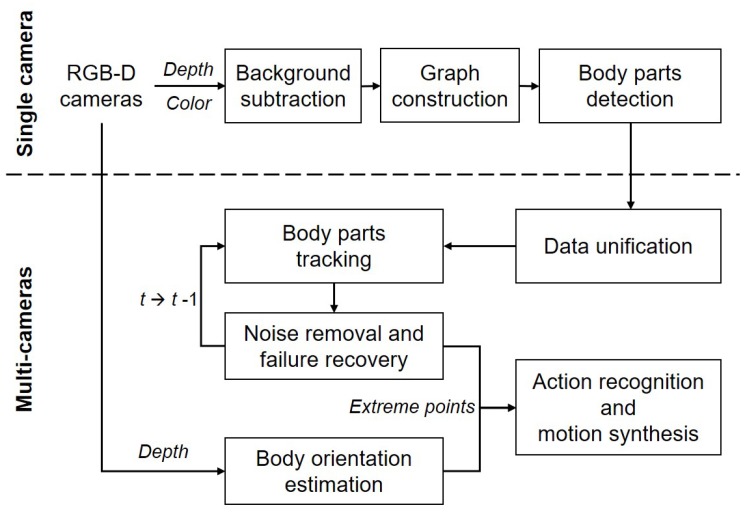
Overview of the proposed system: Human pose estimation using multiple red, green, blue, and depth (RGB-D) cameras.

In this paper, we introduce a marker-less system for human pose estimation by detecting and tracking key body parts, namely, the head, hands, and feet. Given color and depth images captured by multiple RGB-D (two Kinect) cameras, our system detects a set of extreme points on the key body parts from each camera (i.e., the single camera process) and tracks them from the unified data received from the multiple cameras (i.e., the multi-camera process) as shown in Figure 1. During the single camera process, a quadtree-based graph model with segmented regions is constructed from the color and depth images received from a single camera. Using geodesic distances on the graph, a set of candidate points is searched and selected as extreme points through a supervised learning model. During the multi-camera process, the optimal extreme points are selected and tracked from the unified data received from the multiple cameras. For a better tracking performance, a Kalman filter-based method is introduced to reduce positional noises and to recover from a failure of tracking extremes.

Unlike the previous approaches, our system does not reconstruct a full skeleton structure from input data. Instead, input poses are abstracted with a small set of extreme points on the key body parts, making the detecting and tracking processes easier without solving the optimization problem for skeleton reconstruction. Using a small set of extremes as input data, our system can be applied to recognize a human action or to synthesize a motion sequence from a few key poses in real time. As demonstrated in the experimental results, our system shows an average of 87% accuracy against the commercial system, which outperforms the multi-Kinects system with more RGB-D cameras used.

The rest of this paper is organized as follows. Previous approaches for human pose estimation are reviewed in Section 2. The detection of key body parts from each camera is described in Section 3, while tracking them from the unified data received from multiple cameras is detailed in Section 4. The experimental results for tracking accuracy, action recognition, and motion synthesis are demonstrated in Section 5. We conclude this paper with a discussion of potential improvements in Section 6.

## 2. Related Work

Detecting and tracking human body parts from sensor data has been actively researched in computer vision and recognition fields. Using single or multiple RGB-D cameras, the majority of the detection approaches can be differentiated into three categories: Generative (aka top-down), discriminative (aka bottom-up), and hybrid.

The generative approaches [12,13,14] rely on a template model of the human body and try to estimate the model parameters that best describe the pose in an input image. Using the iterative closest point (ICP) algorithm, Grest et al. [12] defined a nonlinear optimization function and estimated a human pose by applying the analytically simplified Jacobian. Based on the probabilistic inferencing algorithm, Zhu et al. [13] performed feature detection on depth images and estimated relatively simple poses from the detected features. Ganapathi et al. [14] performed real-time detection from a sequence of depth images based on the probabilistic temporal model. In their approach, a set of physical and free space constraints are derived to deform the template model. Shuai et al. [15] used multiple depth cameras to minimize occlusion and designed an ellipsoid-based skeleton model to capture the geometry detail of a tracked object. In these ICP-based approaches, the external template model and its parameters need to be configured in advance to initiate the tracking process, which is computationally expensive for a complicated model such as a human body. On the other hand, our system uses no template model and its parameters to track human poses.

The discriminative approaches [4,16,17,18,19] try to detect the body parts directly from the observed data without using an initialization process with a template model. Shotton et al. [4] estimated a list of 3D joint positions on a single depth image by performing per-pixel classification which uses a randomized decision tree with a large image set. Their approach was further exploited by Girshick et al. [16], in which they anticipated the occluded joint positions using the regression forest with relative 3D offset information. Shen et al. [17] introduced an example-based approach which corrects occluded body parts, such as a side view. In their approach, a regression forest is learned based on the differences between the motion capture and Kinect data. Recently, Jung et al. [18] improved the performance of joint estimation using a random tree walk, while Shafaei and Little [19] improved the joint estimation accuracy by applying a convolutional neural network (CNN) based pixel classification. Using the discriminative approaches, the joint positions can be estimated in real time given a large set of high quality data for the training process. For example, Shotton et al. [4] trained each tree with 300,000 images while Shafaei and Little [19] collected a six million data set for their classification. However, our approach searches for a set of key body parts from the hierarchical graph structure using a much smaller set of samples (i.e., less than 1000).

Given a database of human motion, the hybrid approaches [20,21,22,23] try to improve the tracking accuracy by combining the generative and discriminative methods (i.e., solving the optimization problems with the database reference). Ganapathi et al. [20] demonstrated an interactive system that estimates body parts in a kinematic chain structure using the hill-climbing method. In their method, a local detector for body parts (i.e., a discriminative model) is used to initiate a tracking failure. Ye et al. [21] stored a set of a 3D skeleton and its mesh data into a database and obtained the optimal pose by matching the point cloud data through the shape refinement process. Baak et al. [22] showed a method of comparing the joint positions at previous frames and the salient body parts extracted from the depth information to search for similar poses. Later, Helten et al. [23] presented a similar approach with a personalized body tracker that improves the lookup accuracy from the regenerated database. Like the discriminative approaches, an extensive set of samples needs to be prepared in advanced for most hybrid approaches to estimate accurate human poses. For example, Ye et al. [21] captured 19,000 samples from a motion capture system, and Baak et al. [22] selected about 25,000 samples. Furthermore, these approaches are sensitive to the physical property of a user such as a body size and require an additional fitting process to track poses from unknown users, making the approaches less applicable to general users. Using multiple cameras, our approach does not require prior body information to track poses from unknown users; hence, it is more applicable to real-time human action recognition and motion synthesis for unspecified individuals.

Recently, multiple depth cameras [5,6,7,8,9,10,11] have been adopted to overcome the joint occlusion problems by using the unified data captured at different view points. Zhang et al. [5] used multiple Kinects for non-skeletal motion data while Kaenchan et al. [6] applied them for a walking analysis. Kitsikidis et al. [7] adopted a hidden conditional random fields (HCRF) classifier to detect patterns in dance motion. With two synchronized cameras, Michel et al. [8] solved an optimization problem using stochastic optimization techniques to track a human body from the depth volume. Moon et al. [9] adopted a Kalman filter framework to combine the multiple depth data to improve the occlusion problem. Recently, Kim et al. [10,11] demonstrated a large scale of multi-Kinects system to capture dynamic motion in dance and martial arts. Most of the time, these approaches rely on the Kinect method [4] to configure the skeleton structure in an articulated model, which often requires an expensive and complicated post process to enhance naturalness in an output pose. On the other hand, our system detects a small set of key body parts and uses them as inputs to refer to the existing motion data for estimating a dynamic human pose.

## 3. Single Camera Process

Our system acquires a continuous sequence of RGB-D images from multiple cameras. For each camera, major body parts are detected through three steps: Background subtraction, quadtree-based graph construction, and part joint detection using accumulative geodesic distances and a local detector. The details of the steps are described in the subsequence sections.

### 3.1. Background Subtraction

In our system, the RGB-D cameras provide a continuous sequence of color and depth images with same resolution, and both images are calibrated. Given a sequence of color and depth images streamed from a single RGB-D camera, the background information is subtracted from the images to isolate a human object based on the depth information as it is robust to the illumination changes. We captured the first frame of the depth sequence, where no human is visible and subtracted it from subsequent frames. For the depth images with a human object, a threshold value (i.e., the minimum depth value for each pixel) is used to distinguish between the background and foreground objects. This simple method is sensitive to background noises and can generate false positives, especially at the edges of the human object [24]. As a post-processing step, a morphological erode operation and Sobel kernels are applied to reduce such false positive areas. We compute approximation of vertical and horizontal derivatives using Sobel kernels and remove noises from depth images based on gradient magnitudes. From a filtered depth image, $I_{D}$, a corresponding color image, $I_{C}$, can be obtained. We refer a filtered image, $I=\{I_{D},I_{C}\}$. This simple technique works well for a low-resolution depth image. However, a more sophisticated method for the background subtraction could be used using a hardware acceleration [25].

### 3.2. Graph Construction

**Figure 2:**
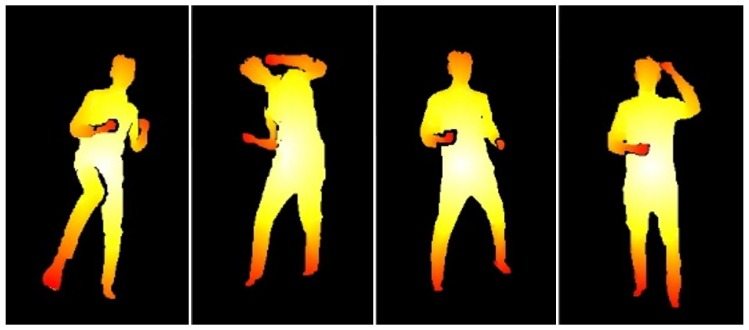
Accumulative geodesic distances between the center of body and key body parts such head, hands, and feet. Here, the poses are colored white if the distances are closer to the center of the body and red if they are closer to key body parts.

Inspired by the work identifying geodesic extreme positions using a graph structure [26,27], our detection method assumes the nature of an invariant body structure such that the accumulative geodesic distances between the center of body and key body parts such as head (H), right hand (RH), left hand (LH), right foot (RF), and left foot (LF) do not change regardless of the human poses as shown in Figure 2. For example, let $P_{C}$ be a center position of the human object averaged from $I_{D}$ and the positions of extreme points, $P_{i}$, on the key body parts are located farthest from $P_{C}$, where $P_{C},P_{i}\in R^{3}$ and i∈{H,RH,LH,RF,LF}. Based on this geodesic characteristic, both $I_{D}$ and $I_{C}$ can be represented as a graph model, *G*. As shown in Algorithm 1, a quadtree-based segmentation is used to group neighboring data efficiently, and each node has a representative value with the center position.

**Algorithm 1** Graph construction: Here, $\delta_{D}$ and $\delta_{C}$ are threshold values to split a node into four children.
1:Input: image I={depth(D),color(C)}, init tree depth $t_{D}=0$2: 3:Output: decomposed image with quadtree structure4: 5:**function**Quadtree($I,t_{D}$)6: 7: **if** ($t_{D}>=t_{\max}$) **then**8: 9:  **return** node(I)10: 11: **else**12: 13:  $\sigma_{D}=$ standard deviation of $I_{D}$14: 15:  $\delta_{D}=$ divider of $I_{D}$16: 17:  $\sigma_{C}=$ standard deviation of $I_{C}$18: 19:  $\delta_{C}=$ divider of $I_{C}$20: 21:  **if** ($\sigma_{D}>\delta_{D}∥\sigma_{C}>\delta_{C}$) **then**22: 23:   $I_{\mathrm{sub}}$ = split *I* as four sub images24: 25:   **return** QuadTree($I_{\mathrm{sub}},t_{D}+1$)26: 27:  **end if**28: 29: **end if**30: 31:
**end function**



### 3.3. Body Parts Detection

Algorithm 1 generates an undirected *G* with a vertex, $v_{j}\in I_{\mathrm{sub}}$, which is a leaf node of the quadtree, and a weighted edge which connects to the neighboring vertices of $v_{j}$. The weight value can be estimated from the Euclidean distance between the neighboring vertices of $v_{j}$.

Let $N_{k}$ be the number of candidate extreme points and $D_{k}$ represent the shortest paths from the *k*th starting vertex, $s_{k}$, where $k=[1,\ldots ,N_{k}]$, to other nodes in *G*. Using Dijkstra’s algorithm, a set of the candidate extreme points, ${\overset{´}{P}}_{k}$, from *G* can be searched in an iterative way as follows,

When k=1,

(1) Set $P_{C}$ as a start vertex, $s_{k}$, and search *G*.
(2) Save the accumulative geodesic distances of (1) to $D_{k}$.
(3) Set ${\overset{´}{P}}_{k}$ to the longest accumulative geodesic end point of $D_{k}$.
(4) Update ${\overset{´}{P}}_{k}$ to $s_{k+1}$.

When $1<k<N_{k}+1$,

(1) Set $s_{k}$ as a start vertex and partially search *G* such that $v_{j}$ is nearer to $s_{k}$ than to $s_{k-1}$.
(2) Update $D_{k-1}$ to $D_{k}$ using the result of (1).
(3) Set ${\overset{´}{P}}_{k}$ to the longest accumulative geodesic end point of $D_{k}$.
(4) Update ${\overset{´}{P}}_{k}$ to $s_{k+1}$.

**Figure 3:**
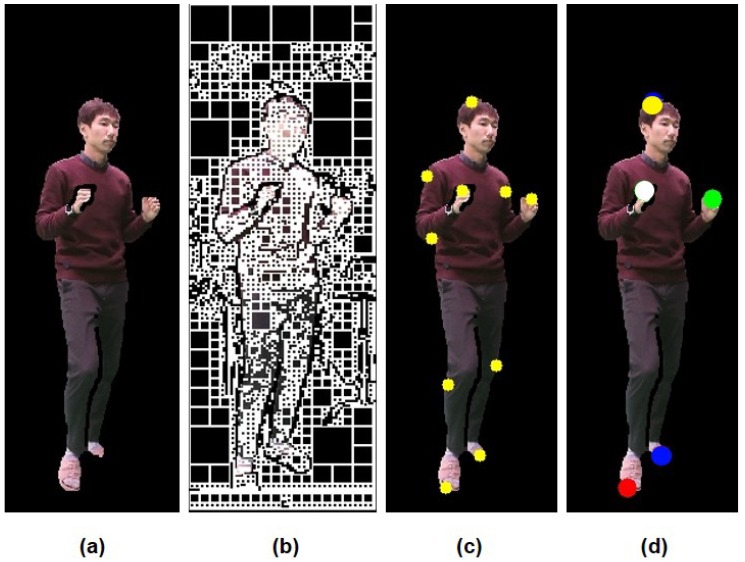
Results of each step in the single camera process: (**a**) a filtered human object (foreground), (**b**) a quadtree-based decomposition of color and depth images, (**c**) accumulative geodesic end points (candidate extreme points), and (**d**) selected extreme points on the head (H) (yellow), right hand (RH) (white), left hand (LH) (green), right foot (RF) (red), and left foot (LF) (blue) in searched regions.

Given ${\overset{´}{P}}_{k}$, $P_{i}$ for the key body parts can be classified by matching local features. The supervised learning model like SVM requires a relatively small amount of sample data and is well suited for the detection of specific human parts [28,29]. For $P_{i}$ classification, the image patches of major joints are collected from $I_{C}$, and data augmentation is used to increase the number of the patches. The histograms of gradients for the patches are arranged into a 1D feature vector and used to train the SVM [30]. During the test process, $P_{i}$ is classified within the region of interest for ${\overset{´}{P}}_{k}$ (i.e., 80 by 80 pixels) by applying a sliding window (i.e., 5 to 20 pixels) with multi-scales for scale-invariant detection. Figure 3 shows the results of each step with $t_{\max}=8$, $\delta_{D}=8$, $\delta_{C}=5$, and $N_{k}=10$ to specify $P_{i}$.

## 4. Multi-Camera Process

Our system combines a set of key body parts detected from each camera into a single space and tracks the body parts with minimum errors. This multi-camera process consists of four steps: Data unification in a single coordinate system, body part tracking based on a voting scheme, noise removal and failure recovery with a Kalman-filtered method, and body orientation estimation using principal component analysis (PCA). The details of each step are described in the subsequent sections.

### 4.1. Data Unification

**Figure 4:**
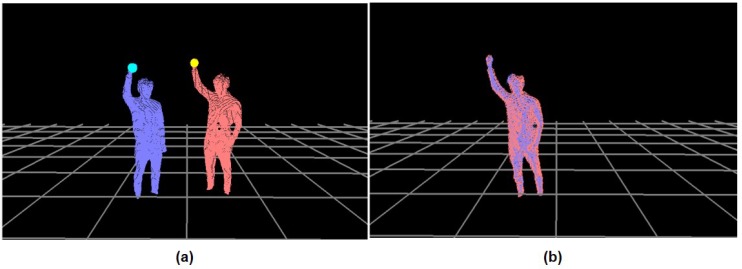
Data unification for a multi-camera process: (**a**) a calibration pose for the coordinate unification and (**b**) two depth images in the same coordinate system (oriented toward a viewer).

Using multiple RGB-D cameras, a set of extreme points, $P_{i}^{c}$, detected from the *c*th camera can be unified into the same coordinate system by using a rigid transformation, **T**. If one of the cameras is selected as a reference coordinate system, **T** can be estimated from the ICP method [31] by minimizing the error function, $E\left(T\right)=E\left(R_{T},L_{T}\right)$, where $R_{T}$ and $L_{T}$ are the rotation and translation of the camera data to the reference system, respectively. This convergence method is capable of an online performance with the input data obtained from multiple cameras; however, the unified result may be erroneous without enough matching points, $M_{t}$, where $M_{t}\in R^{3}$ and $t\in [1,\ldots ,N_{t}]$. As shown in Figure 4, $P_{\mathrm{RH}}$ of the calibration pose is traced from the reference, $M_{t}^{R}$, and the *c*th camera, $M_{t}^{c}$, respectively. Given $M_{t}$, $E(R_{T},L_{T})$ can be evaluated as follows:

(1) $$E\left(R_{T},L_{T}\right)\propto \frac{1}{N_{t}}\sum_{t=1}^{N_{t}}\left|\right|M_{t}^{R}-R_{B}\left(R_{T}M_{t}^{c}+L_{T}\right){\left|\right|}^{2}.$$

Here, $L_{T}={\bar{M}}^{R}-R_{T}{\bar{M}}^{c}$, where ${\bar{M}}^{R}=\frac{1}{N_{t}}\sum_{t=1}^{N_{t}}M_{t}^{R}$ and ${\bar{M}}^{c}=\frac{1}{N_{t}}\sum_{t=1}^{N_{t}}M_{t}^{c}$. Given a correlation matrix, **W**, between $M_{t}^{R}$ and $M_{t}^{c}$,

(2) $$W=\sum_{t=1}^{N_{t}}\left(M_{t}^{R}-{\bar{M}}_{t}^{R}\right){\left(M_{t}^{c}-{\bar{M}}_{t}^{c}\right)}^{⊤}={UCV}^{⊤}.$$

Here, the optimal solution for $E(R_{T},L_{T})$ is $R_{T}={UV}^{⊤}$ with $W={UCV}^{⊤}$ derived from a single value decomposition (SVD). Furthermore, $R_{B}$ is the body rotation between the depth images from the reference camera, $I_{D}^{R}$, and the *c*th camera, $I_{D}^{c}$. This is estimated by a plane defined by the upper three extremes, namely, $P_{H},P_{\mathrm{LH}},$ and $P_{\mathrm{RH}}$ and used to enhance the ICP performance. In our system, $M_{t}$ is collected at every 33 ms until $N_{t}=500$.

### 4.2. Body Parts Tracking

Given a set of extreme points for each body part in a single coordinate system, our tracking method uses a voting scheme to set a priority for each point. At first, a set of $P_{i}^{c}$ for each body part *i* from the *c*th camera forms a candidate group. Within this group, the distance between $P_{i}^{c}$ below a threshold value (150 mm) is regarded as the same point and counted as a vote, $\nu_{i}^{D}$. Next, the characteristics of human physical constraints are considered using the accumulative geodesic distance for the vote count. For example, starting from $P_{C}$, the end joints such as head, hands, and feet are generally located further than the internal joints such as neck, elbows, and knees. Similarly, the accumulative geodesic distances from $P_{C}$ to the end joints are longer than ones to the internal joints. Another vote, $\nu_{i}^{R}$, is counted for the point if the distance from $P_{C}$ is larger than another threshold value (a quarter of user’s height). A total vote, $\nu_{i}$, for $P_{i}^{c}$ is counted as follows:

(3) $$\nu_{i}=w_{i}^{D}\nu_{i}^{D}+w_{i}^{R}\nu_{i}^{R}.$$

Here, $\nu_{i}^{D}$ and $\nu_{i}^{R}$, where $\nu_{i}^{D},\nu_{i}^{R}\in \left[0,1\right]$, are the vote counts from the distance measure and the range measure, respectively. Furthermore, $w_{i}^{D}$ and $w_{i}^{R}$ are the weight values for $\nu_{i}^{D}$ and $\nu_{i}^{R}$, respectively. In our system, $w_{i}^{D}$ and $w_{i}^{R}$ are set to 2 and 1 to emphasize the importance of the neighboring factor, $\nu_{i}^{D}$.

Once $\nu_{i}$ is counted for $P_{i}^{c}$, $P_{i,t}$ is tracked based on the minimum Euclidean distance between a tracked point at a previous frame, $P_{i,t-1}$, and candidate points at a current frame, $P_{i,t}^{c}$, by maximizing $\nu_{i}$ as follows:

(4) $$P_{i,t}=\underset{P_{i,t}^{c}}{\arg\min}{∥\langle P_{i,t}^{c},\nu_{i}\rangle -P_{i,t-1}∥}^{2},$$

where $\langle P_{i,t}^{c},\nu_{i}\rangle$ is the extreme point from the *c*th camera at the *t* frame with $\nu_{i}$ votes. Here, $P_{i,t}^{c}$ is compared to $P_{i,t-1}$ in order from the largest $\nu_{i}$ to the smallest one. If the maximum of $\nu_{i}$ is 0, the tracking attempt fails and enters a recovery process described in the following section.

### 4.3. Noise Removal and Failure Recovery

Whenever the tracking process fails or causes positional noises such as jerky movements in a trajectory of $P_{i,t}$, our system applies a Kalman filter-based method to correct the erroneous $P_{i,t}$. Assuming a linear system used for a state-space model in a Kalman filter, the system state model and its measurement model can be defined as follows:

(5) $$\begin{array}{c} x_{t+1}={Ax}_{t}+w_{t},\\ z_{t}={Hx}_{t}+v_{t}, \end{array}$$

where *t* is the time index, **A** is the state transition model, $x_{t}$ is the state vector, $w_{t}$ is the state noise vector, **H** is the measurement matrix, $z_{t}$ is the measurement vector, and $v_{t}$ is the measurement noise vector. Here, $w_{t}$ and $v_{t}$ are considered to be white noises, which comply to the Gaussian normal distribution with a mean value of 0, a covariance matrix of $Q={\mathrm{ww}}^{⊤}$, and $R={\mathrm{vv}}^{⊤}$. As input arguments to $x_{t}$, the position and velocity of $P_{i,t}$ are used, and $z_{t}$ returns a corrected position of $P_{i,t}$. In our system, $\sigma^{2}$ in **Q** and **R** is set to 0.01 and 1.0, respectively.

Given the state-space model, the Kalman filter estimates a predicted position, ${\tilde{P}}_{i,t}$ from the prediction and correction steps with $P_{i,t}$. For example, the prediction step estimates ${\tilde{P}}_{i,t}$ while the correction step removes the noises in $P_{i,t}$. During the prediction step, a predicted state vector, ${\tilde{x}}_{t}$, and a predicted covariance matrix, ${\tilde{P}}_{t}$, are estimated from a posteriori at t−1 as follows:

(6) $$\begin{array}{c} {\tilde{x}}_{t}={Ax}_{t-1},\\ {\tilde{P}}_{t}={AP}_{t-1}A^{⊤}+Q, \end{array}$$

where $x_{t-1}$ and $P_{t-1}$ are the posteriori state estimate and the posteriori error covariance matrix at time t−1, respectively. Here, ${\tilde{x}}_{t}$ replaces ${\tilde{P}}_{i,t}$, which failed to be located during the tracking process. During the correction step, ${\tilde{x}}_{t}$ and the Kalman gain matrix, $K_{t}$, are used to update $x_{t}$ as follows:

(7) $$\begin{array}{c} K_{t}={\bar{P}}_{t}H^{⊤}{\left(H{\tilde{P}}_{t}H^{⊤}+R\right)}^{-1},\\ {\dot{x}}_{t}={\tilde{x}}_{t}+K_{t}\left(P_{i,t}-H{\tilde{x}}_{t}\right). \end{array}$$

**Figure 5:**
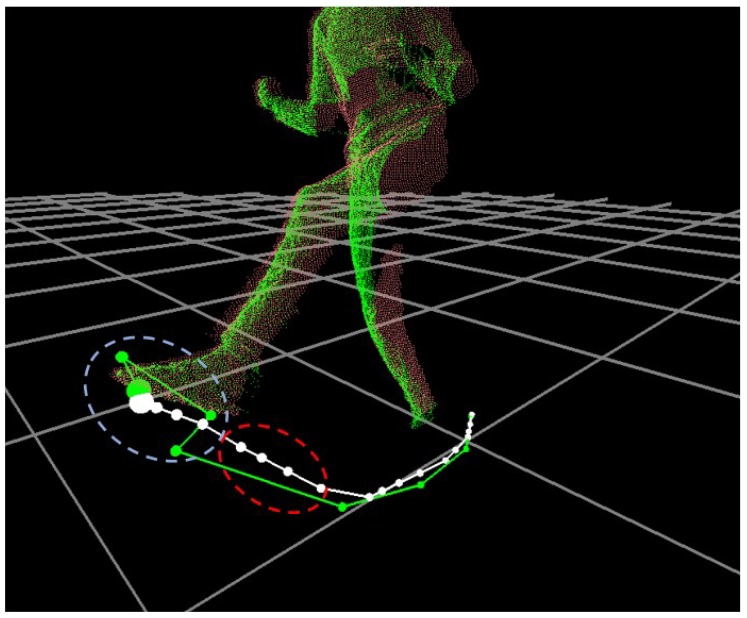
Comparison of foot trajectories: Initially tracked (green) and Kalman filtered (white). Here, the tracking noises cause sudden positional changes (the white circle) while the tracking failure skips the foot positions (the red circle), which are corrected in the filtered trajectory.

Here, ${\dot{x}}_{t}$ is the updated state vector, which removes the noises and sets a corrected position of $P_{i,t}$. Finally, the posteriori error covariance matrix at time *t*, $P_{t}$, is estimated as follows:

(8) $$P_{t}={\tilde{P}}_{t}-K_{t}H{\tilde{P}}_{t},$$

which will be used during the prediction step at t+1. To summarize, ${\tilde{x}}_{t}$ from the prediction step and ${\dot{x}}_{t}$ from the correction step determines $P_{i,t}$ which fails to track or needs to be corrected for its position, respectively. The result of this process for $P_{\mathrm{LF},t}$ is shown in Figure 5.

### 4.4. Body Orientation Estimation

**Figure 6:**
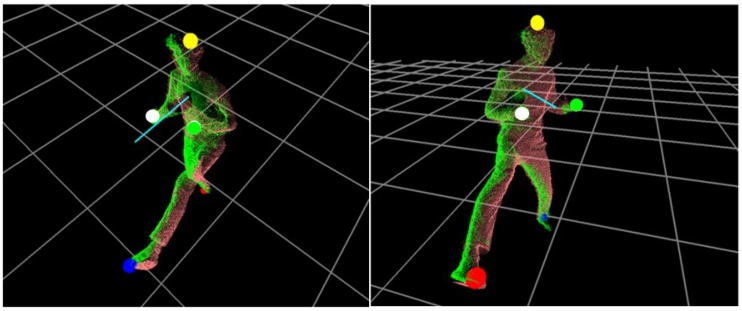
A set of extreme points on the body parts tracked and the body orientation represented by a normal vector (cyan).

The body orientation of each pose from $I_{D}$, serve as a useful parameter for motion analysis. In our system, the normal vector at $P_{C}$, $n_{C}$, is estimated using PCA, which finds the best fitting plane from the point locations in $I_{D}$. When PCA is applied to the selected locations in $I_{D}$, the first two eigenvectors define the plane. For example, when a covariance matrix (i.e., a size of 3 by 3) is estimated for the matrix of coordinates from $I_{D}$ (i.e., a size of $N_{s}$ by 3), where $N_{s}$ is the number of points to be fit, it can be decomposed into a set of eigenvectors and eigenvalues. Here, the first two eigenvectors with the largest eigenvalues define a plane; thus, the cross product of these two eigenvectors defines a normal vector (i.e., body orientation), $n_{D}$, on the plane. Figure 6 shows a set of $P_{i}$ tracked and a body orientation represented by $n_{C}$. In our system, $n_{C}$ is placed 300 mm higher from $P_{C}$ for better recognition.

## 5. Experimental Results

**Figure 7:**
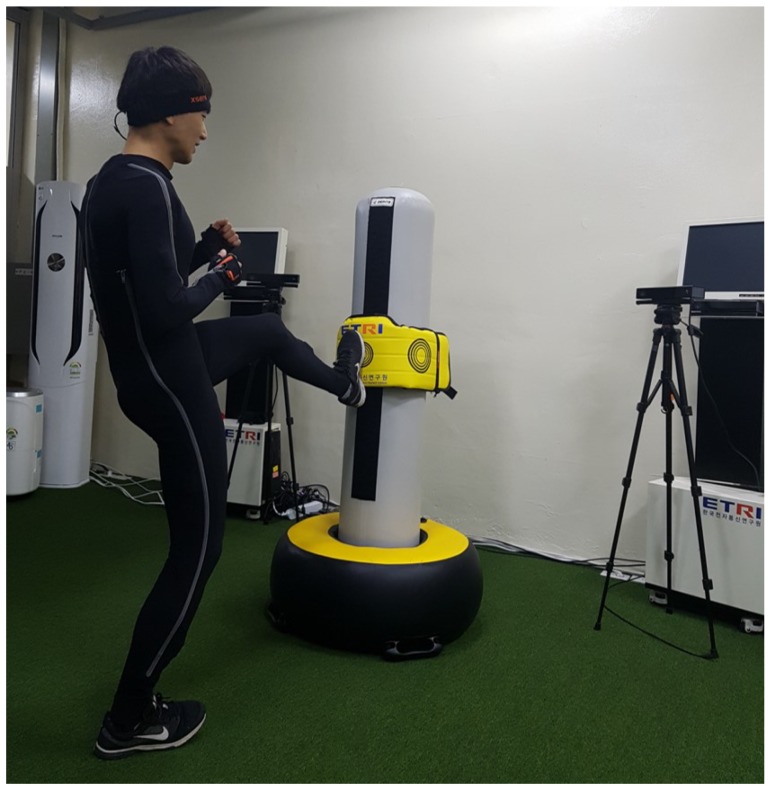
System setup for action recognition and motion synthesis: Two RGB-D cameras with displays, a smart sandbag to detect user kicks and punches, and the Xsens system (a wearable suit) used for accuracy comparison.

Figure 7 shows the prototype of our system, which tracks various dynamic movements of Taekwondo from general users. In this system, two RGB-D (Kinect v2 [2]) cameras (Microsoft, Redmond, WA, USA) are placed in front of the user with two displays. From the cameras, only RGB and depth data are retrieved to track a set of extreme points on key body parts such as head, hands, and feet, and the body orientation. A smart sandbag, equipped with pressure sensors, is self-manufactured and used to detect the hitting moments such as punches and kicks from input motion. Our system is best understood through examples of its uses, as described in following sections, and the accompanying video (located at https://drive.google.com/open?id=1IJjt0TTs0TimcEcsTSoIbWrxZiiNYv9g).

### 5.1. Tracking Accuracy

To evaluate the tracking accuracy between different systems, the ground-truth data are captured by the Xsens system [32]. As shown in Figure 7, this commercial system uses a set of inertial sensors embedded in a wearable suit such that user motions can be tracked from both camera-based and Xsens systems at the same time. Owing to the differences in the sensing locations on key body parts between the two systems, a set of joint vectors, j, is defined from the center of the body to each of the end-effector joints (i.e., hands and feet) and used to compare the angular differences between two outputs as follows:

(9) $$Err=1-\frac{\sum_{0}^{N_{a}}\left(j_{X}\cdot j_{C}+1\right)}{2N_{a}},$$

where $j_{C}$ and $j_{X}$ are measured with a total of $N_{a}$ frames from the camera-based and Xsens system, respectively.

**Figure 8:**
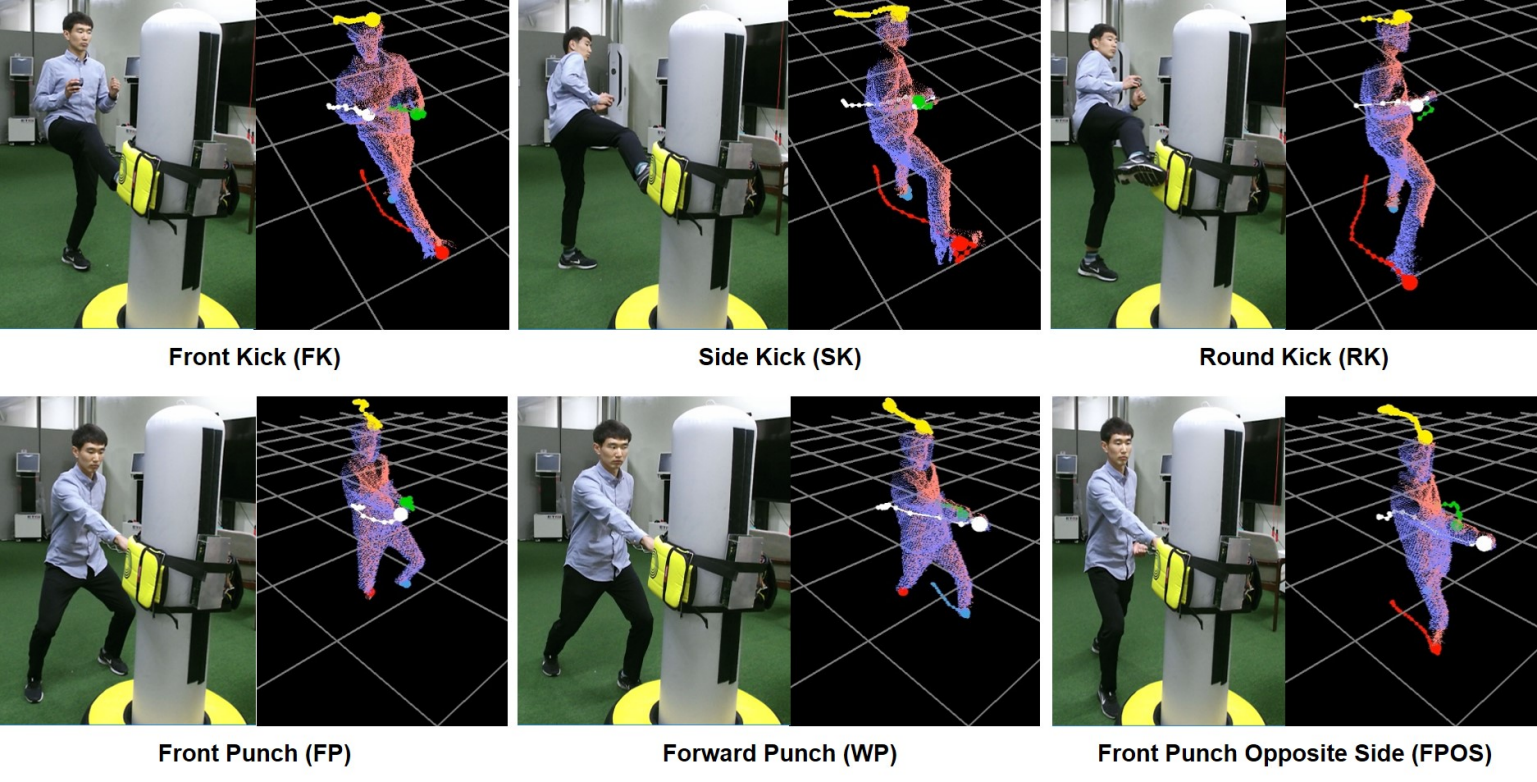
Recognition of various Taekwondo actions: Input RGB (**left**) and output depth data with tracked extreme points (**right**).

For this and next comparisons, a total of 3845 frames are collected from six Taekwondo actions in Figure 8. As shown in Table 1, it is notable that the head part shows the highest accuracy, which is visible most of time during the tracking process. However, the hand parts are less accurate than the head and foot parts. This is mainly because larger noises arise in the hand areas whenever a user takes poses with hands on the torso areas. These poses are frequent in Taekwondo actions in Figure 8 and make the local detection ambiguous between the hand and torso areas.

Next, our system is compared against the multi-Kinects system [11]. In this comparison, the body parts tracked by our system are compared against the major joints (i.e., *HEAD, HAND_RIGHT, HAND_LEFT, ANKLE_RIGHT, ANKLE_LEFT*) recovered from the Kinect skeletal data. As shown in Table 2, our system with two Kinect cameras outperforms the multi-Kinects system with two or four cameras throughout all of tracked body parts. As expected, the head part shows the highest accuracy as it is visible most of time from all cameras. However, for other parts, the multi-Kinects system suffers from the erroneous skeleton reconstruction, especially the foot areas, and shows relatively less accuracy. It is noteworthy that using four cameras for our system shows negligible improvements on tracking accuracy due to the majority of frontal movements in the collected data.

### 5.2. Action Recognition

As shown in Figure 8, our system recognizes various kick and punch motions from a user through three phases:Iinput motion segmentation, feature vector extraction, and motion type recognition. First, an input motion is segmented by detecting starting and ending moments of key poses. The starting moment is determined based on the speed and position of the hands and feet. For example, an input motion starts at a moment when the speed of the hands and feet are under a threshold value (1 m/s) while the positions of both feet stay under a height threshold value (10% of the user’s height). The motion ends at the moment when the sandbag system detects a hit from the user. To recognize a motion piece by comparing it to a reference, these motions should be aligned in the time-space domain as each of the segmented motions differ in temporal length and the user’s body size. Using a number of samples (10 to 15 depending on the complexity of the input poses), a sample set, $P_{i,t}^{S}$ and $n_{C,t}^{S}$, are linearly interpolated from the trajectories of $P_{i,t}$ and $n_{C,t}$ in the segmented motion and defines the feature vector. For a normalization of the feature vector, $P_{i,t}^{S}$ is translated to an origin using the average positions between $P_{i,0}^{S}$ of a foot and dividing them by the user’s height. In addition, $n_{C,t}^{S}$ is normalized by 360 degrees.

Given the feature vector for each input motion, a SVM is utilized to recognize different motion types. Table 3 shows training and test sets (a total of 67,925 frames from 1465 training and 392 test samples) used for recognizing 12 types of Taekwondo motions in an offline manner. For the data set, 10 general users performed each motion type. Table 4 shows that our system is capable of recognizing the motion types with over an average of 96% accuracy from the test set. However, some of the similar motions are incorrectly tracked and misclassified from the test set. For example, about 9% of a front punch with the right hand was recognized as a front punch with a left hand due to the fast exchanges of the left and right hands from user input motions.

### 5.3. Motion Synthesis

**Figure 9:**
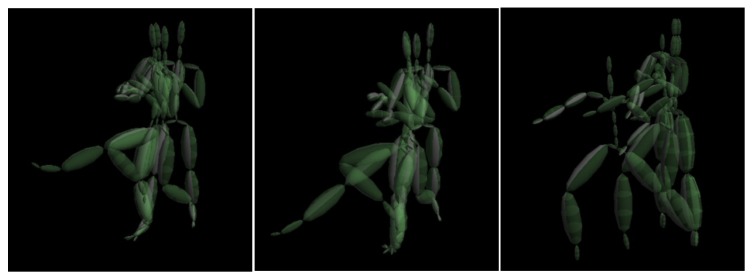
Key poses used for motion synthesis: front kick (**left**), round kick (**middle**), and front punch (**right**).

As shown in Figure 9, our system is tested for synthesizing a sequence of dynamic movements from a few key poses. The key poses are captured from the Xsens system. Provided with a set of key poses with input parameters (a set of extremes tracked from our system), a sequence of in-between poses between the keys can be generated from the motion blending technique with their weight values estimated from the multi-dimensional scattered interpolation [33].

**Figure 10:**
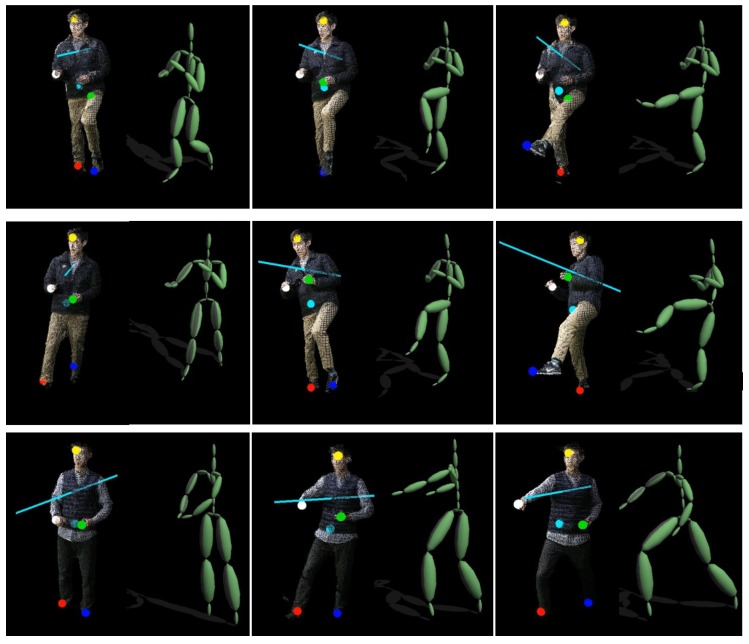
Motion synthesis from a set of key poses and input parameters: Front kick (**top**), round kick (**middle**), and front punch (**bottom**).

Figure 10 shows an instance of synthesizing three Taekwondo motions, where each of them is generated using a set of tracked extremes as input parameters and blending five key poses from example motions. As demonstrated in the results, the synthesized motions are comparable to the input motions, exhibiting key movements of each Taekwondo motion type. It is noteworthy that some of movement details, such as relative hand and foot positions, are not synthesized in the output motions due to the small number of key poses used to generate the in-between poses. In these results, it took about 3.43 s, 2.98 s, and 5.78 s to synthesize 1406, 1335, and 2485 frames, respectively. Thus, our system can produce over 400 frames per second, showing a real-time performance for motion synthesis.

## 6. Conclusions

In this paper, we introduced a marker-less system for human pose estimation by detecting and tracking key body parts: Head, hands, and feet. Using multiple RGB-D cameras, our system minimizes the self-occlusion problems by unifying the depth data captured at different viewpoints into a single coordinate system. To accelerate the search process of the candidate points on the body parts, a quadtree-based graph is constructed from the RGB-D image, and the accumulative geodesic distances on the graph is used to select a set of extreme points on the key body parts. During the tracking process, these points are used as input parameters for motion analysis. Whenever there are tracking noises or failures, a Kalman filter-based method for noise removal and recovery is introduced to correct and expect the extreme positions. Unlike the previous approaches using a learning-based model, our approach does not reconstruct a full skeleton structure to estimate human poses from input data. Instead, the input poses are abstracted with a small set of extreme points, making the detecting and tracking process easier without solving the optimization problem for skeleton reconstruction. Using a small set of extremes as input data, our system can be applied to recognize a human action or to synthesize a motion sequence from a few key poses in real time. As demonstrated in the experimental results, our system shows a higher accuracy over the multi-Kinects system with more RGB-D cameras used.

The current system can be easily scalable by adding more RGB-D cameras as needed. For example, placing two more RGB-D cameras behind the user might provide better accuracy for the occluded poses in turning motion if the space and system cost are permitted. Using other RGB-D camera such as Intel RealSense [3] was problematic due to noisy depth data and unstable support for the software library. Furthermore, our system is mainly designed to capture one user at a time. For the multi-person pose estimation, the current detection method can be exploited to extract multiple independent keys, possibly other than hands and feet, from the input images and to map each set of the keys to a different person.

The proposed system causes higher tracking errors when there are frequent crossing of hands and feet in an input pose. We are currently improving the tracking recovery process of such cases by analyzing the velocity gradients of hands and feet. In addition, there is no synchronization in the times between the input data received from multiple Kinect cameras that do not support a triggering signal. An external sync generator can be adopted with more sophisticated cameras; however, such a configuration increases the overall system cost, making the system less applicable for general users.

## Figures and Tables

**Table 1:** Tracking accuracy of our system.

Action Type		Accuracy (%)				
		H	RH	LH	RF	LF
Front Kick (FK)	Left	92.1	88.2	87.8	86.4	87.8
	Right	91.8	87.8	88.1	87.1	86.9
Side Kick (SK)	Left	90.2	87.9	88.0	87.8	88.3
	Right	90.8	87.7	87.9	89.1	87.1
Round Kick (RK)	Left	91.8	88.1	88.2	87.2	89.2
	Right	91.5	88.3	88.1	88.9	87.9
Front Punch (FP)	Left	94.2	81.8	84.1	85.6	85.8
	Right	94.1	83.8	82.7	86.1	86.2
Forward Punch (WP)	Left	93.8	80.9	82.8	86.2	86.5
	Right	94.0	83.1	80.5	86.1	86.4
Front Punch Opposite Side (FPOS)	Left	93.8	80.2	82.7	85.8	86.0
	Right	93.1	83.8	80.9	85.4	85.9
Average		92.6	85.1	85.2	86.8	87.0

**Table 2:** Tracking accuracy comparison between our system and multi-Kinects system [10].

System Type	Average Accuracy (%)				
	H	RH	LH	RF	LF
Ours (Two Kinects)	92.6	85.1	85.2	86.8	87.0
Multi-Kinects (Two Kinects)	91.2	78.9	79.2	76.5	77.1
Multi-Kinects (Four Kinects)	91.3	80.3	80.8	79.8	80.0

**Table 3:** Training and test sets used for action recognition in Taekwondo. The first column represents the action type and motion side to be recognized. A total of 1465 and 392 samples are collected from 10 general users to train (the second column) and to test (the third column) the action recognition, respectively. Here, the total frames for each action (the fourth column) combine the frames used in the training and test samples while each action has a different temporal length (the last column).

Action Type		Training Samples	Test Samples	Total Frames	Average Frames (σ)
FK	Left	123	34	4160	26.50 (±3.37)
	Right	120	34	4488	29.14 (±6.84)
SK	Left	123	30	4886	31.93 (±5.48)
	Right	123	30	5305	34.67 (±4.41)
RK	Left	124	37	3470	21.55 (±3.68)
	Right	125	35	4403	27.52 (±5.82)
FP	Left	120	33	4758	31.10 (±6.22)
	Right	120	32	7617	32.16 (±4.45)
WP	Left	124	30	6243	40.54 (±4.33)
	Right	121	32	6380	41.01 (±4.14)
FPOS	Left	121	31	7617	50.11 (±7.20)
	Right	121	34	8598	55.47 (±5.79)
Total		1465	392	67,925	

**Table 4:** Accuracy of action recognition in Taekwondo.

Action Type		FK		SK		RK		FP		WP		FPOS	
		Left	Right	Left	Right	Left	Right	Left	Right	Left	Right	Left	Right
FK	Left	0.97	0	0.03	0	0	0	0	0	0	0	0	0
	Right	0	0.94	0	0.06	0	0	0	0	0	0	0	0
SK	Left	0.03	0.03	0.94	0	0	0	0	0	0	0	0	0
	Right	0	0.03	0	0.97	0	0	0	0	0	0	0	0
RK	Left	0	0	0	0	1.00	0	0	0	0	0	0	0
	Right	0.03	0	0	0	0	0.97	0	0	0	0	0	0
FP	Left	0	0	0	0	0	0	1.00	0	0	0	0	0
	Right	0	0	0	0	0	0	0.09	0.91	0	0	0	0
WP	Left	0	0	0	0	0	0	0	0	0.97	0	0	0.03
	Right	0	0	0	0	0	0	0	0	0	0.97	0.03	0
FPOS	Left	0	0	0	0	0	0	0	0	0	0	1.00	0
	Right	0	0	0	0	0	0	0	0	0	0	0	1.00

## Abbreviations

The following abbreviations are used in this manuscript:

RGB-D	Red, green, blue, and depth
HCI	Human computer interaction
VR	Virtual reality
AR	Augmented reality
AdaBoost	Adaptive boosting
SVM	Support vector machine
GMM	Gaussian mixture model
ICP	Iterative closest point
CNN	Convolutional neural network
H	Head
RH	Right hand
LH	Left hand
RF	Right foot
LF	Left foot
SVD	Single value decomposition
PCA	Principal component analysis
FK	Front kick
SK	Side kick
RK	Round kick
FP	Front punch
WP	Forward punch
FPOS	Front punch opposite side
